# Comparative Study of Tomato and Tomato Paste Supplementation on the Level of Serum Lipids and Lipoproteins Levels in Rats Fed With High Cholesterol

**DOI:** 10.5812/ircmj.1007

**Published:** 2013-04-05

**Authors:** Mir Hadi Khayat Nouri, Ali Namvaran Abbas Abad

**Affiliations:** 1 Department of Pharmacology and Toxicology, Veterinary Faculty, Islamic Azad University, Tabriz Branch, Tabriz, IR Iran; 2 Young Researchers Club, Islamic Azad University, Tabriz Branch, Tabriz, IR Iran

**Keywords:** Hypercholesterolemia, Lipoproteins, Lycopersicon Esculentum, Rats

## Abstract

**Background:**

Hypercholesterolemia is one of the risk factors of cardiovascular diseases. Increased blood cholesterol affects general health and increases mortality due to cardiovascular disease. Poor nutrition increases LDL cholesterol and decreases LDL receptor activities in the liver. Scientists have shown that consumption of antioxidants can reduce hypercholesterolemia and proved benefits of fruit and vegetables. Tomato reduces oxidative stress by increasing serum total antioxidant level.

**Objectives:**

This study compared the tomato and tomato paste supplementation on the level of serum lipids and lipoproteins in rats fed with high cholesterol.

**Materials and Methods:**

In this study, four male rat groups (10 rats per group) were used. Control group received basal diet, second group received basal diet and 2% cholesterol (Chol), third and fourth groups received basal diet, 2% cholesterol tomato and tomato paste respectively (20 percent of the diet) for a month. Then serum TC, LDL, HDL and TG were measured.

**Results:**

Results showed that in Chol group, all lipids increased significantly (P < 0.05) except HDL compared to the control group. Tomato and tomato paste supplementation decreased TC, LDL and TG concentration significantly (P < 0.05) compared to Chol group. Tomato paste had the higher effect on lipids decreasing than tomato.

**Conclusions:**

Decreases of TC, LDL and TG may be related to tomato antioxidant effect. This course in human required more investigations.

## 1. Background

Hyperlipidemia is one of the causes of death from cardiovascular diseases. Increase of blood cholesterol could treat public health (1, 2). Hypercholesterolemia is observed in most industrial societies that its main reason is poor nutrition with food containing saturated fats and high cholesterol. Poor nutrition with high cholesterol increases the cholesterol level, LDL and triglycerides levels. On the other hand, hypercholesterolemia decreases LDL receptors activity in the liver. Studies showed that increase of triglyceride and cholesterol levels could decrease blood HDL level (3). Increased serum LDL level and decreased HDL level, are the main factors involved in cardiovascular diseases, especially coronary atherosclerosis, the development that causes inflammation and reduces the endothelial function and wide vascular lesions (4). Oxidation of LDL Lipoprotein in vessels increases progression of cardiovascular diseases, but increased serum HDL (unlike LDL) can prevent progress of hypercholesterolemia and cardiovascular diseases (5, 6). Atherosclerosis is one of the diseases that can be caused by various factors (Multifactorial Diseases). The incidence of the disorder contributes genetic factors and environmental effects and their interaction can affect their changes. The symptoms of their interaction appear with serum lipids and lipoproteins changes, which eventually cause heart arterial diseases (1, 4, 6, 7). Studies indicate that changes in blood concentrations of lipids and lipoproteins, including triglyceride (TC), high density lipoprotein (HDL), low density lipoprotein (LDL) and total cholesterol (TC) are involved in atherosclerosis; therefore they are used in diagnosis of this type of diseases. Also it has been shown that increased serum cholesterol and LDL on one hand and decreased HDL levels on the other hand are primary factors for predicting atherosclerosis and cardiovascular diseases (1, 4, 6, 7). Results of studies have shown that consumption of antioxidant nutrients and some food decreases the occurrence of cardiovascular diseases due to hypercholesterolemia in humans and experimental animals (8-14). Tomato’s scientific name is Solanum lycopersicum or lycopersicon esculentum and its general name is Tomato. Nowadays tomato is used raw or cooked as sauce and tomato paste. Compounds and substances in ripe tomato constitutes about 80 percent water and the remaining are protein, fat, sugars types including glucose and fructose, vitamins A, C, K, E, thiamin, riboflavin, pantothenic acid, folic acid, almost all essential amino acids, minerals including calcium, phosphorus, iron, sodium, potassium, magnesium, copper, manganese, cobalt, zinc, arsenic and iodine (15). Original and major color materials of tomato are carotenoid, beta-carotene and lycopene. Many pigments within fruits and vegetables have antioxidant effects, so body can use these materials for scavenging free radicals (13, 16-24). Lycopene, that causes redness in tomatoes, is a pigment from carotenoid family. Lycopene is the most abundant carotenoid found in tomatoes and ripe tomatoes and its value increases between 10 and 14 times. Some types of carotenoids can be converted to vitamin A after consumption, but lycopene does not have this property and form approximately 50 % of carotenoids which comprise human serum. Otherwise, by adequate diet, the deposits decrease rapidly (13, 16-24). Studies have shown that lycopene found in tomatoes due to intensive antioxidant properties decreases the risk of cancer. Tomato consumption prevents pancreatic, lung, prostate and uterus cancer (21). Tomatoes consist of C and E vitamins and each of these vitamins has antioxidant properties (13, 17-19, 24, 25). In addition to above mentioned materials, phenolic and flavonoids compounds are found in tomato, these compounds, due to their antioxidant properties, have beneficial therapy which effects on many diseases (17, 22, 24-32). In traditional medicine, tomato has been used for relief and recovering from some diseases such as asthma, cough, flu, eye diseases, ear pain, typhoid, yellow fever, colitis, arthritis (33), decreasing blood glucose and cholesterol (16, 34, 35), regulating blood pressure and reducing heart diseases (36). Several reactions in body cause peroxide, which leads to oxidizing other molecules such as proteins or nucleic acids. Oxidized molecules lose their normal function and in many cases they find malicious functions. To deal with these reactions and their harmful consequences, other molecules and mechanisms help body defend the event called antioxidant. Any reason that causes increase of pro- oxidant is called oxidative stress. In this case, the field is provided for preventing many diseases such as cardiovascular problems following diabetes disease. Therefore it appears that nutritional received antioxidant substances reduce oxidative stress, and play an important role in prevention of diabetes and cardiovascular diseases. Results of studies have shown that Lycopene increases the rate of body antioxidants. Also tomato prevents LDL from converting to harmful oxidized LDL and prevents arteries plaque (13, 16-24, 36, 37).

## 2. Objectives

The aim of this study was to investigate the effect of supplementation with tomato and tomato paste on serum levels of Lipids and lipoproteins in male rats fed with high cholesterol.

## 3. Materials and Methods

In this study, 40 male Wistar rats (for each group n = 10) were used. Weight of selected animals was 250-300 g, and their age was 10 weeks. Animals were acclimated to the laboratory environment for 5-7 days before being used in the study. Animals were kept in a humid environment under a 12-hour light/dark cycle (lights on at 7 AM). Food and water were available ad lib. The National Institutes of Health guidelines for care and use of animals and Guidelines on Ethical Standards for Investigation of Experimental in Animals were followed. All efforts were made to minimize the number of animals under study and their suffering degree. Animals were randomly divided into four groups. One group as control group (receiving basal diet), the second group received three subsequent dietary which contains cholesterol 2% ration daily for 30 days (7, 19, 38), and third and fourth groups received 20% tomato and tomato paste diet daily for a month. At the end of 30 days, after a day of fasting, all animals were anesthetized by ether and then blood samples were taken from the animals. Samples were centrifuged for preparing blood serum. Total Cholesterol (TC), High Density Lipoprotein (HDL) and Triglyceride (TG) were measured by auto analyzer from isolated serum samples. Low density lipoprotein (LDL) was calculated by using the Friedewald-Fredrickson formula as mg/dl (LDL = TC-HDL-TG/5) (39). SPSS 13th version software was used to analyze data. Group data were presented as mean ± SEM and analyzed statistically by using One-Way ANOVA (ANOVA) followed by Tukey multiple comparison tests. The level for statistical significance was set at a P value of < 0.05.

## 4. Results

Measurement of serum TC, LDL, HDL and TG in tested groups showed that the measure of TC, LDL and TG in the group received dietary fat (Chol) increased significantly (P < 0.05) and the amount of HDL decreased significantly (P < 0.05) in comparison to the control group (Table 1). The amount of TC, LDL and TG decreased significantly (P < 0.05), but the amount of HDL increased significantly (P < 0.05) in rats fed with high fat diet with tomato supplements (tomato + Chol) and tomato paste (tomato paste + Chol) compared to Chol group, it shows that there were no significant differences in control group (Table 1). Comparative study showed that tomato paste had decreasing effect on lipids significantly (Table 1).

**Table 1.** Effect of Supplementation with Tomato and Tomato Paste on TC, LDL, HDL and TG (mg/dl) in Serum of Male Rats Fed with High Cholesterol Diet

Groups (n = 10), Mean ± SEM	TG^a^	HDL^a^	LDL^a^	TC^a^
**Control**	45.6 ± 1.25	43.7±1.4	38.2±1.24	90.8±1.85
**Chol^a^**	98.7 ± 2.1^b^	33.2±1.44^b^	58.3±1.85^b^	112.5±1.91^b^
**tomato + Chol**	52.59 ± 2.59^c^	41.16±1.3^c^	40.22±1.37^c^	91.6±1.45^c^
**Tomato paste + Chol**	48.44 ± 1.75^c^	42.78±1.9^c^	39.22±1.17^c^	90.4±1.22^c^

^a^Abbreviations: Chol, high cholesterol diet; HDL, high density lipoprotein; LDL, low-density lipoprotein; TC, total cholesterol; TG, triglycerides

^b^P < 0.05 compared with the control group

^c^P < 0.05 compared with Chol is in each column

## 5. Discussion

In this study, the effect of supplementation with tomato and tomato paste on high cholesterol diet induced hyperlipidemia in male rats was studied. Studies indicate that changes in blood concentrations of lipids and lipoproteins, including triglyceride, HDL, LDL and cholesterol are observed in atherosclerosis. It has been shown that increased serum cholesterol and LDL on one hand and decreased HDL levels on the other hand are primary factors for predicting atherosclerosis and cardiovascular diseases (1, 4, 6, 7). As the results of this study showed, adding 2 percent of cholesterol in dry matter intake for one month can alter blood lipids concentration. Adding cholesterol to rats’ diet increased the concentration of total cholesterol, LDL and TG but decreased HDL level. These results are consistent with the results sought by Gorinstein et al. (1998) and Vaskonen et al. (2002) and it showed that the amount of TC and LDL increased in hypercholesterolemic rats (7, 38). This model is used widely to create animal models of diet-induced hypercholesterolemia with high cholesterol and fat. In this study, the tomato and tomato paste decreased amount of TC, LDL and TG and increased HDL levels. Various researchers have shown the effect of oral administration of this substance on blood parameters. So the results of this study confirm the results of Ibrahim et al. (2008), Ali et al. in rat (17, 40). Studies of Ibrahim et al. (2008) on three different tomato products including powder, paste and tomato catch up sauce and comparing their effects on body weight, lipid profiles, liver enzymes and the atherogenic index of rats reported that these three products have significantly beneficial therapeutic effects on rats and beneficial effects were mostly related to the tomato paste and catch up sauce (40). Ali et al. (2009) showed that lycopene extracted from tomatoes is able to reduce concentrations of glucose, hydrogen peroxide, serum lipids and increase insulin concentrations, catalase, superoxide dismutase and glutathione peroxidase following the use of Streptozotocin in rats (17) Gitenay et al. (2007) showed that tomatoes decrease the effect of triglyceride and antioxidant levels more than lycopene which effects on oxidative stress induced by vitamin E deficiency in rats. Also tomato increases superoxide dismutase enzyme levels of red blood cells more than lycopene (26) Bobek et al. (1998) studied the effects of dried fruits like applea, grapes and tomato on rats, tomato decreased 15% of cholesterol and Hydroxy-Methyl-Glutaryl-CoA enzyme (HMG-CoA) but increased serum antioxidant enzymes to 56% (41). Bobek (1999) also confirmed the previous report which found that dried tomato reduced cholesterol, VLDL and LDL to 24% but increased HDL to 26%. In liver, activities of Superoxide Dismutase enzyme and Glutathione Peroxidase enzyme increased (42). Fujiwara et al. (2007) showed that Esculeogenin A; as a type of glycoside in the tomato; can decrease cholesterol, triglyceride, LDL significantly and reduce atherosclerotic lesions in ApoE (Apolipoprotein E) deficient mice. They described these effects as Cholesterol Acyltransferase which inhibits the protein function (43). Hsu et al. (2008) showed that administration of tomato paste in 3-9 percent of dietary level can decrease cholesterol and LDL levels to 14.3% and 11.3% respectively in the hamster. They also reported that after eight weeks of tomato paste consumption, HDL level increases up to 28.8%. They subsequently reported that administration of tomato paste decreased Malondialdehyde to 89.33%, and increases Superoxide Dismutase, Catalase and Glutathione Peroxidase enzyme activities in more than groups received high fat diet. Their proposed mechanism on the antioxidant effect of lycopene was related to Carbonic Anhydrase III (CAIII) and Adenylate Kinase II (AK2) (27). Unlike the above studies, Fredrikson et al. (2007) showed that lycopene can prevent the incidence of hypercholesterolemia, oxidation of plasma lipids and aortic atherosclerotic changes 16 weeks after lycopene consumption extracted from tomato in the Watanabe Heritable hyperlipidemic rabbits (44). Several reports presented the effect of tomato on human. In a study by Ahuja et al. (2006) on 21 individuals with age of 22-70 years old , they compared tomato lycopene and olive oil and showed that both compounds increased HDL and decreased cholesterol and triglyceride levels, may decrease cardiovascular diseases due to the effects on serum lipid profiles (16). Rein et al. (2006) studied the effect of tomato flavonoids on some risk factors for cardiovascular diseases and showed that these substances reduced C-reactive protein (CRP) and fibrinogen but in contrast increased vitamin E-selenium and serum HDL (30). In a study on 32 women and 16 men for one month with high tomato (300 g daily) diet, Blum et al. (2006) showed that diet significantly increased the serum HDL (15.2 %). They also reported that cholesterol, triglyceride, LDL and VLDL have been decreased, but these changes were not significant (34). Bose et al. (2006) showed that in type II diabetic patients with long-term administration of tomato, lycopene reduced Glycosylated Hemoglobin (HbA1c), Malondialdehyde, Triglyceride, LDL, VLDL and cholesterol but in contrast increased antioxidant enzymes like Superoxide Dismutase (SOD) and Glutathione Peroxidase (GSH-Px) (19). In 2007, they also confirmed the previous results and reported that lycopene decreased lipid profiles except HDL, increased antioxidant levels and decreased lipid peroxidation in cardiovascular patients (45). Jacob et al. (2008) suggested that the antioxidant and tomato protective effects are not only due to lycopene but also) regardless of the role of vitamin C, lycopene cannot be related to tomato effects (25). Upritchard et al. (2000) studied on 57 patients with type II diabetes and showed that tomato juice increased LDL oxidation time, and decreased plasma glucose, C-reactive protein (CRP) and adhesion molecules circulation. They also, based on their results, suggested that the antioxidant effect is very important in myocardial infarction prevention in diabetic patients (24). Wang et al. (2006) studied on 35,783 women in the USA and reported that the uses of lycopene or lycopene-rich foods like tomatoes care not related to the incidence of diseases such as diabetes type II, cancer and cardiovascular diseases (46). Collins et al. (2004) also confirmed the results in middle-aged women (20). However, Basu et al. (2007) reported that tomato as lycopene source, and foods like tomato juice, tomato paste, tomato sauce and catch-up can be a source for antioxidants in the body, preventing the incidence of diseases associated with Oxidative stress. Diseases that Basu et al. mentioned are Diabetes mellitus type II, prostate cancer and cardiovascular diseases and they noted that tomato and lycopene reduce plasma lipoprotein levels, DNA damage, oxidative stress and prostate specific antigen (PSA) (18). Reboul et al. (2005) reported the beneficial effect of lycopene and tomato on prostate cancer (37). By this explanation, the difference between sexes may be realized. In summary, this study showed that supplementation of tomato and tomato paste associated with high cholesterol diet in rats, decreased the amount of TC, LDL and TG but increased HDL concentration. These effects can be due to the antioxidant and specific compounds which constituent tomatoes, and probably function by inhibiting lipid peroxidation and decrease the production of cholesterol, LDL and triglycerides. However, the role of tomato and tomato paste as a supplement for the prevention of hypercholesterolemia in humans, need further investigation.

## References

[1] Verschuren WM, Jacobs DR, Bloemberg BP, Kromhout D, Menotti A, Aravanis C (1995). Serum total cholesterol and long-term coronary heart disease mortality in different cultures. Twenty-five-year follow-up of the seven countries study.. JAMA..

[2] (2001). Executive Summary of The Third Report of The National Cholesterol Education Program (NCEP) Expert Panel on Detection, Evaluation, And Treatment of High Blood Cholesterol In Adults (Adult Treatment Panel III).. JAMA..

[3] Grundy SM, Denke MA (1990). Dietary influences on serum lipids and lipoproteins.. J Lipid Res..

[4] Ross R (1993). The pathogenesis of atherosclerosis: a perspective for the 1990s.. Nature..

[5] Devlin TM (1991). Text book of biochemistry with clinical correlations..

[6] Malloy MJ, Kane JP, Katzung BG (2004). Agents used in hyperlipidemia.. Basic and clinical pharmacology..

[7] Vaskonen T, Mervaala E, Krogerus L, Karppanen H (2002). Supplementation of plant sterols and minerals benefits obese Zucker rats fed an atherogenic diet.. J Nutr..

[8] Chorvathova V, Bobek P, Ginter E, Klvanova J (1993). Effect of the oyster fungus on glycaemia and cholesterolaemia in rats with insulin-dependent diabetes.. Physiol Res..

[9] Fukushima M, Nakano M, Morii Y, Ohashi T, Fujiwara Y, Sonoyama K (2000). Hepatic LDL receptor mRNA in rats is increased by dietary mushroom (Agaricus bisporus) fiber and sugar beet fiber.. J Nutr..

[10] Hong L, Xun M, Wutong W (2007). Anti-diabetic effect of an alpha-glucan from fruit body of maitake (Grifola frondosa) on KK-Ay mice.. J Pharm Pharmacol..

[11] Hu SH, Liang ZC, Chia YC, Lien JL, Chen KS, Lee MY (2006). Antihyperlipidemic and antioxidant effects of extracts from Pleurotus citrinopileatus.. J Agric Food Chem..

[12] Kabir Y, Yamaguchi M, Kimura S (1987). Effect of shiitake (Lentinus edodes) and maitake (Grifola frondosa) mushrooms on blood pressure and plasma lipids of spontaneously hypertensive rats.. J Nutr Sci Vitaminol (Tokyo)..

[13] Wang HX, Ooi VE, Ng TB, Chiu KW, Chang ST (1996). Hypotensive and vasorelaxing activities of a lectin from the edible mushroom Tricholoma mongolicum.. Pharmacol Toxicol..

[14] Yoshioka Y, Tabeta R, Saito H, Uehara N, Fukuoka F (1985). Antitumor polysaccharides from P. ostreatus (Fr.) Quel.: isolation and structure of a beta-glucan.. Carbohydr Res..

[15] Rice RP, Rice LW, Tindall HD (1994). Fruit and vegetable production in warm climates..

[16] Ahuja KD, Pittaway JK, Ball MJ (2006). Effects of olive oil and tomato lycopene combination on serum lycopene, lipid profile, and lipid oxidation.. Nutrition..

[17] Ali MM, Agha FG (2009). Amelioration of streptozotocin-induced diabetes mellitus, oxidative stress and dyslipidemia in rats by tomato extract lycopene.. Scand J Clin Lab Invest..

[18] Basu A, Imrhan V (2007). Tomatoes versus lycopene in oxidative stress and carcinogenesis: conclusions from clinical trials.. Eur J Clin Nutr..

[19] Bose KS, Agrawal BK (2006). Effect of long term supplementation of tomatoes (cooked) on levels of antioxidant enzymes, lipid peroxidation rate, lipid profile and glycated haemoglobin in Type 2 diabetes mellitus.. West Indian Med J..

[20] Collins JK, Arjmandi BH, Claypool PL, Perkins-Veazie P, Baker RA, Clevidence BA (2004). Lycopene from two food sources does not affect antioxidant or cholesterol status of middle-aged adults.. Nutr J..

[21] Heber D, Lu QY (2002). Overview of mechanisms of action of lycopene.. Exp Biol Med (Maywood)..

[22] Rao AV (2002). Lycopene, tomatoes, and the prevention of coronary heart disease.. Exp Biol Med (Maywood)..

[23] Silaste ML, Alfthan G, Aro A, Kesaniemi YA, Horkko S (2007). Tomato juice decreases LDL cholesterol levels and increases LDL resistance to oxidation.. Br J Nutr..

[24] Upritchard JE, Sutherland WH, Mann JI (2000). Effect of supplementation with tomato juice, vitamin E, and vitamin C on LDL oxidation and products of inflammatory activity in type 2 diabetes.. Diabetes Care..

[25] Jacob K, Periago MJ, Bohm V, Berruezo GR (2008). Influence of lycopene and vitamin C from tomato juice on biomarkers of oxidative stress and inflammation.. Br J Nutr..

[26] Gitenay D, Lyan B, Rambeau M, Mazur A, Rock E (2007). Comparison of lycopene and tomato effects on biomarkers of oxidative stress in vitamin E deficient rats.. Eur J Nutr..

[27] Hsu YM, Lai CH, Chang CY, Fan CT, Chen CT, Wu CH (2008). Characterizing the lipid-lowering effects and antioxidant mechanisms of tomato paste.. Biosci Biotechnol Biochem..

[28] Lean ME, Noroozi M, Kelly I, Burns J, Talwar D, Sattar N (1999). Dietary flavonols protect diabetic human lymphocytes against oxidative damage to DNA.. Diabetes..

[29] Noroozi M, Burns J, Crozier A, Kelly IE, Lean ME (2000). Prediction of dietary flavonol consumption from fasting plasma concentration or urinary excretion.. Eur J Clin Nutr..

[30] Rein D, Schijlen E, Kooistra T, Herbers K, Verschuren L, Hall R (2006). Transgenic flavonoid tomato intake reduces C-reactive protein in human C-reactive protein transgenic mice more than wild-type tomato.. J Nutr..

[31] Saxena R, Venkaiah K, Anitha P, Venu L, Raghunath M (2007). Antioxidant activity of commonly consumed plant foods of India: contribution of their phenolic content.. Int J Food Sci Nutr..

[32] Shen YC, Chen SL, Wang CK (2007). Contribution of tomato phenolics to antioxidation and down-regulation of blood lipids.. J Agric Food Chem..

[33] Lazarus SA, Bowen K, Garg ML (2004). Tomato juice and platelet aggregation in type 2 diabetes.. JAMA..

[34] Blum A, Merei M, Karem A, Blum N, Ben-Arzi S, Wirsansky I (2006). Effects of tomatoes on the lipid profile.. Clin Invest Med..

[35] Kordella T (2005). You say tomato..same blood glucose, different A1Cs. Research profile. Stuart A Chalew, MD.. Diabetes Forecast..

[36] (2005). Tomato juice may protect against heart disease.. Health News..

[37] Reboul E, Borel P, Mikail C, Abou L, Charbonnier M, Caris-Veyrat C (2005). Enrichment of tomato paste with 6% tomato peel increases lycopene and beta-carotene bioavailability in men.. J Nutr..

[38] Gorinstein S, Bartnikowska E, Kulasek G, Zemser M, Trakhtenberg S (1998). Dietary persimmon improves lipid metabolism in rats fed diets containing cholesterol.. J Nutr..

[39] Friedewald WT, Levy RI, Fredrickson DS (1972). Estimation of the concentration of low-density lipoprotein cholesterol in plasma, without use of the preparative ultracentrifuge.. Clin Chem..

[40] Ibrahim HS, Ahmed LA, El-din MM (2008). The functional role of some tomato products on lipid profile and liver function in adult rats.. J Med Food..

[41] Bobek P, Ozdin L, Hromadova M (1998). The effect of dried tomato, grape and apple pomace on the cholesterol metabolism and antioxidative enzymatic system in rats with hypercholesterolemia.. Nahrung..

[42] Bobek P (1999). Dietary tomato and grape pomace in rats: effect on lipids in serum and liver, and on antioxidant status.. Br J Biomed Sci..

[43] Fujiwara Y, Kiyota N, Hori M, Matsushita S, Iijima Y, Aoki K (2007). Esculeogenin A, a new tomato sapogenol, ameliorates hyperlipidemia and atherosclerosis in ApoE-deficient mice by inhibiting ACAT.. Arterioscler Thromb Vasc Biol..

[44] Frederiksen H, Rasmussen SE, Schroder M, Bysted A, Jakobsen J, Frandsen H (2007). Dietary supplementation with an extract of lycopene-rich tomatoes does not reduce atherosclerosis in Watanabe Heritable Hyperlipidemic rabbits.. Br J Nutr..

[45] Bose KS, Agrawal BK (2007). Effect of lycopene from cooked tomatoes on serum antioxidant enzymes, lipid peroxidation rate and lipid profile in coronary heart disease.. Singapore Med J..

[46] Wang L, Liu S, Manson JE, Gaziano JM, Buring JE, Sesso HD (2006). The consumption of lycopene and tomato-based food products is not associated with the risk of type 2 diabetes in women.. J Nutr..

